# Repeatability and reproducibility of multiparametric magnetic resonance imaging of the liver

**DOI:** 10.1371/journal.pone.0214921

**Published:** 2019-04-10

**Authors:** Velicia Bachtiar, Matthew D. Kelly, Henry R. Wilman, Jaco Jacobs, Rexford Newbould, Catherine J. Kelly, Michael L. Gyngell, Katherine E. Groves, Andy McKay, Amy H. Herlihy, Carolina C. Fernandes, Mark Halberstadt, Marion Maguire, Naomi Jayaratne, Sophia Linden, Stefan Neubauer, Rajarshi Banerjee

**Affiliations:** 1 Perspectum Diagnostics Ltd, Oxford, United Kingdom; 2 Department of Life Sciences, University of Westminster, London, United Kingdom; 3 Oxford Centre for Clinical Magnetic Resonance Research, Radcliffe Department of Medicine, University of Oxford, Oxford United Kingdom; Linköping University, SWEDEN

## Abstract

As the burden of liver disease reaches epidemic levels, there is a high unmet medical need to develop robust, accurate and reproducible non-invasive methods to quantify liver tissue characteristics for use in clinical development and ultimately in clinical practice. This prospective cross-sectional study systematically examines the repeatability and reproducibility of iron-corrected T1 (cT1), T2*, and hepatic proton density fat fraction (PDFF) quantification with multiparametric MRI across different field strengths, scanner manufacturers and models. 61 adult participants with mixed liver disease aetiology and those without any history of liver disease underwent multiparametric MRI on combinations of 5 scanner models from two manufacturers (Siemens and Philips) at different field strengths (1.5T and 3T). We report high repeatability and reproducibility across different field strengths, manufacturers, and scanner models in standardized cT1 (repeatability CoV: 1.7%, bias -7.5ms, 95% LoA of -53.6 ms to 38.5 ms; reproducibility CoV 3.3%, bias 6.5 ms, 95% LoA of -76.3 to 89.2 ms) and T2* (repeatability CoV: 5.5%, bias -0.18 ms, 95% LoA -5.41 to 5.05 ms; reproducibility CoV 6.6%, bias -1.7 ms, 95% LoA -6.61 to 3.15 ms) in human measurements. PDFF repeatability (0.8%) and reproducibility (0.75%) coefficients showed high precision of this metric. Similar precision was observed in phantom measurements. Inspection of the ICC model indicated that most of the variance in cT1 could be accounted for by study participants (ICC = 0.91), with minimal contribution from technical differences. We demonstrate that multiparametric MRI is a non-invasive, repeatable and reproducible method for quantifying liver tissue characteristics across manufacturers (Philips and Siemens) and field strengths (1.5T and 3T).

## Introduction

As the burden of non-alcoholic fatty liver disease (NAFLD) reaches epidemic levels in developed countries [1], [2], there is a pressing need to develop non-invasive, standardised, and quantitative methods [3]. Liver biopsy has long been the gold standard for staging liver disease, yet it is painful, prone to sampling variability [4], has poor inter-observer concordance [5] and carries a risk of complications [6]. Magnetic Resonance Imaging (MRI)-based methods are attractive as they are sensitive to subtle differences in tissue composition, can sample the entire liver, and yield objective quantitative measurements that can contribute to prospective patient management [7]–[9].

Multiparametric MRI is a safe and non-invasive method for quantification of liver tissue characteristics. Images for quantification of hepatic fat from proton density fat fraction (PDFF) maps, T2*, and iron-corrected T1 (cT1) can be rapidly obtained during abdominal breath-hold acquisitions without the need for contrast agents or additional external hardware [8], [10]. Iron correction of T1 (cT1) is necessary to address the confounding effects of excess iron, which is common in chronic liver disease. Liver *MultiScan* (LMS, Perspectum Diagnostics, Oxford, UK) is a software application that can be used with supported MR-systems to correct T1 for the effects of excess iron, and thus, to calculate cT1 from T1 and T2* maps, and standardise to a 3T field strength [10]. This method has been shown to have high diagnostic accuracy for the assessment of liver fibrosis compared to histology [8], predict clinical outcomes in patients with mixed liver disease aetiology [7], identify patients with non-alcoholic steatohepatitis (NASH) and cirrhosis [9], reliably excludes clinically significant liver disease with superior negative predictive value (83.3%) to liver stiffness (42.9%) and is cost-effective in diagnosing NAFLD [11], [12]. Additionally, a recent two-centre study showed excellent test-retest reliability for multiparametric MRI derived metrics (CoV range of 1.4% to 2.8% for cT1) in 22 healthy volunteers [13], indicating good technical precision of this method.

The reliability, or precision of metrics are defined as the extent to which measurements can be reproduced under different conditions such as different scanner field strengths, manufacturers, and models (reproducibility), and reflects the degree of agreement between repeated measurements under identical or near-identical conditions (scan-rescan repeatability) [14]. To be clinically useful, metrics also need to be effective in measuring the heterogeneity of physiological and pathological values in the population [15]. The ability to standardise a measurement across different MR scanner field strengths, manufacturers and models is particularly relevant in the context of clinical practice and multi-site clinical trials.

The purpose of this study was to systematically test the repeatability, reproducibility, and intra- and inter-operator reliability of cT1, T2*, and PDFF measurements across scanner field strength, manufacturer, and model in human participants and phantoms. The performance of T1-mapping standardisation was also evaluated in phantoms.

## Materials and methods

### Study design and population

**Fig 1:**
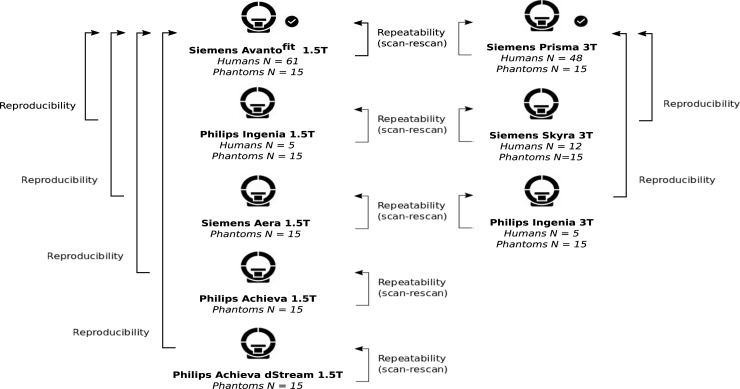
Study design. Two manufacturers (Siemens and Philips) and a range of scanner models were used to systematically test the repeatability and reproducibility of multiparametric-MRI derived measurements in human participants and phantoms.

Sixty-one participants (aged 22–80, mean 42 years; 25 males; BMI 18–39, mean 25) gave their written informed consent to participate. This study received ethical approval from the South Central Oxford Research Ethics Committee C (Ref: 17/SC/0205). Participants included those with mixed liver disease aetiology (n = 32) and those without any history of liver disease (n = 29) in order to represent a wide range of values of hepatic fat, iron, and fibro-inflammatory status. Exclusion criteria included the presence of MRI contraindications and the inability to obtain informed consent. MR operators and data analysts were blinded to the indication of participants with liver disease and those without. All participants underwent two serial multiparametric MRI examinations per scanner on at least two different scanners in pseudorandomised order (Fig 1). Same scanner scan-rescan were done on the same day and the time between different scanners ranged from same-day up to 1 week. Participants were instructed to take nothing by mouth for 4 hours before their scan time.

### Phantom multiparametric MRI

Phantoms were manufactured to span the normal and clinically relevant range of values expected in the liver to reflect the heterogeneity within the population of interest [16]. Three phantoms, each specific to T1, T2*, and PDFF were manufactured. T1 phantoms were agar gel-based using NiCl_2_ as the paramagnetic relaxation modifier (range: 338-1075ms at 1.5T and 351-1137ms at 3T). T2* phantoms were aqueous solutions of MnCl_2_ (range: 3-70ms at 1.5T and 2-43ms at 3T). PDFF phantoms were peanut oil and agar gel-based (0–100% at 1.5T and 3T) manufactured according to the methods of Hines and colleagues [16] (Sigma-Aldrich, UK).

Phantoms were scanned on four Siemens (Avanto^fit^ 1.5T, E11C, MyoMaps; Prisma 3T, E11C, MyoMaps; Skyra 3T, E11C, MyoMaps; Siemens Healthineers) and four Philips (Ingenia 1.5T, 5.3.0, CardiacQuant; Ingenia 3T, 5.3.0, CardiacQuant; Achieva 1.5T, R5.3, CardiacQuant; Achieva dStream 1.5T, R5.3, CardiacQuant; Philips Healthcare) scanners. MyoMaps for Siemens systems and CardiacQuant for Philips systems are commercially available modified Look-Locker inversion recovery (MOLLI) T1-mapping sequences [17]. All phantom measurements were performed with a simulated ECG triggering at 1 beat/s. Differences in the MRI sequences used on Siemens and Philips platforms produce systematic differences in fitted T1 values. These quantitative differences were resolved by using distinct, separately acquired phantom measurements to generate linear mapping functions to standardise the values obtained on one system to those from another at the same nominal magnetic field strength (1.5T or 3T). All 3T systems were linearly mapped to the Siemens Prisma 3T, and all 1.5T systems to the Siemens Avanto^fit^ 1.5T, defined as the reference scanners, see Supporting Information S2 File.

### Human multiparametric MRI

All human MR scans were performed with participants lying supine on three Siemens (Avanto^fit^ 1.5T, E11C, MyoMaps, OCMR Oxford; Prisma 3T, E11C, MyoMaps, OCMR Oxford; Skyra 3T, E11C, MyoMaps, Southampton General Hospital, UK; Siemens Healthineers) and two Philips (Ingenia 1.5T, 5.3.0, CardiacQuant, Leiden University Medical Centre; Ingenia 3T, 5.3.0, CardiacQuant, Leiden University Medical Centre; Philips Healthcare) scanners. Local radiographers at each imaging centre were trained on the protocol and performed the scans in this study. Single transverse slices were captured through the centre of the liver through the porta hepatis. The individual components of the multiparametric MR protocol consist of T1, T2*, and PDFF-mapping. Full details of the scanning sequences for each scanning platform can be found in Supporting Information S1 File. Linear mappings to reference scanners were performed in the same manner as phantoms, as described above. Any bias introduced by elevated iron was removed from the T1-measurements, yielding the iron-corrected T1 (cT1) as previously described [8], [10]. All human scans on both field strengths used the Siemens Prisma 3T as the reference scanner.

### Image processing

Anonymised MR data were analysed off-site using Liver *MultiScan* software (Version 2, Perspectum Diagnostics, UK). Image analysts were trained in abdominal anatomy and images with artefacts were referred to a team of experienced MR physicists for evaluation as previously described [18]. Out of the 138 scans that were completed, 3 scans were unquantifiable due to acquisition errors and in 7 instances due to problems with the scanner cooling system (unrelated to this study and protocol), resulting in a scan success rate of 93%. For each acquisition, three 15mm diameter circular regions of interest (ROIs) were selected on the transverse cT1, T2*, and PDFF maps to cover a representative sample of the liver parenchyma. To assess intra-reader variability, analyst 1 (AN1) re-measured the values for all participants and scan repeats in a randomised order. The time between re-reads was greater than 1 day. To examine inter-reader variability, the first read from analyst 1 (AN1) was compared to an independent read from analyst 2 (AN2). Analysts were blinded to all participant and scanner information.

### Statistical analysis

Statistical analyses were carried out using R 3.1.1 [19]. The Bland-Altman method was used to investigate the repeatability and reproducibility between different scanner models against the reference scanners for each metric in phantom (T1, T2*, and PDFF) and human (cT1, T2*, and PDFF) measurements. Repeatability (scan-rescan) was assessed as the closeness of agreement using identical equipment (same scanner field strength, manufacturer, and model). Reproducibility was assessed as the closeness of agreement under varying circumstances (different scanner field strength, manufacturer, and model), such as would be encountered in a multi-centre setting. Limits of Agreement were calculated as the mean of the differences plus and minus 1.96 times the standard deviation of the differences. Repeatability and reproducibility coefficients are reported as 1.96 times the standard deviation of the differences. Mean coefficients of variation are the mean of the coefficients of variation for each individual.

To further interrogate the reliability of the cT1 metric, a Linear Mixed Effects (LME) approach was implemented using the nlme package [20] in R [19]. LME modelling has been demonstrated to be a superior method to common alternatives such as repeated measures ANOVA or simple paired students t-test as it provides greater statistical power and is robust in the face of missing data [21]. Importantly, LME models for replication data separately and effectively model variance due to within and between subject factors [15], [22]. To assess the variance that could be accounted for by each explanatory variable, the intraclass correlation coefficient (ICC) was calculated to determine the proportion of the total variability in the observations that is due to the differences between pairs.

## Results

### Standardisation of phantom measurements

**Fig 2:**
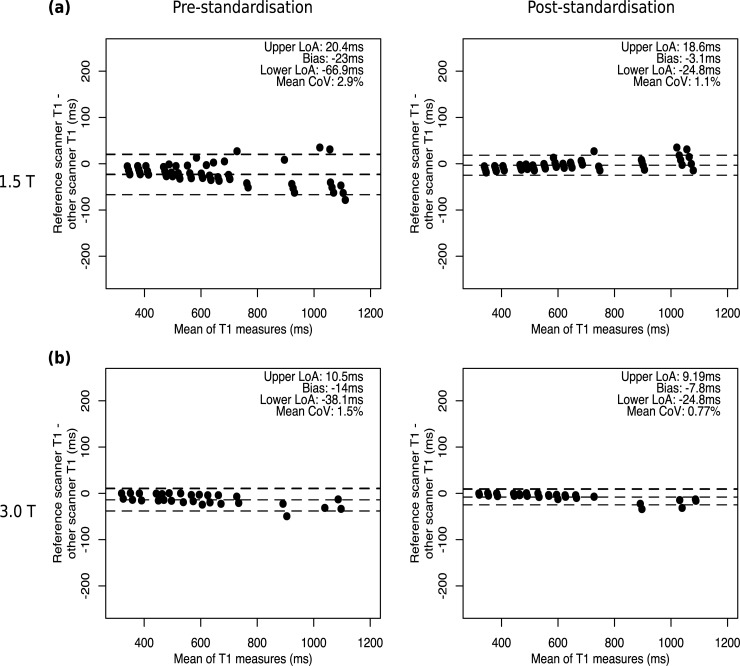
Phantom T1 Standardisation. Bland-Altman plots demonstrating T1 measurements in phantoms before and after standardisation at **(a)** 1.5T and **(b)** 3T.

We tested the performance of the standardisation of T1 maps across different scanner field strengths, manufacturers, and models using phantom measurements. Bland-Altman analysis of phantom-derived mappings from 90 acquisitions across scanner models and software versions before and after standardization (Fig 2) showed a clear reduction in bias (1.5T: from -23ms to -3.1ms; 3T: from -14ms to -7.8ms), tightening of the 95% Limits of Agreement (LoA) (1.5T: from -66.9ms– 20.4ms, to -24.8ms– 18.6ms; 3T: from -38.1ms– 10.5ms, to -24.8ms– 9.19ms) and a decrease in the mean coefficient of variation (CoV) (1.5T: 1.5% to 0.77%; 3T: 2.9% to 1.1%).

### Repeatability and reproducibility of phantom measurements

**Fig 3:**
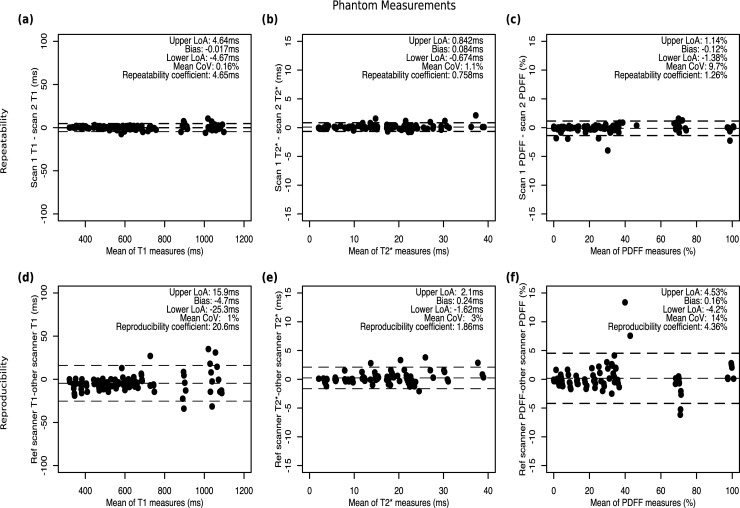
Repeatability and reproducibility of phantom measurements. Bland-Altman plots from phantom measurements across manufacturer and field strength for **(a)** T1, **(b)** T2*, and **(c)** PDFF.

Standardized T1 from phantom-derived mappings demonstrated high repeatability (CoV 0.16%, bias -0.02 ms, 95% LoA of -4.7 to 4.7 ms) and reproducibility (CoV 1%, bias -4.7 ms, 95% LoA of -25.3 ms to 15.9 ms). T2*-mappings showed good repeatability (CoV 1.1%, bias 0.08 ms, 95% LoA of -0.67 to 0.84 ms) and reproducibility (CoV 3%, bias 0.24ms, 95% LoA of -1.62ms to 2.1ms). Similarly, PDFF measurements also showed good repeatability (CoV 9.7%, bias -0.12%, 95% LoA of -1.4 to 1.14%) and reproducibility (CoV 14%, bias 0.16%, 95% LoA of -4.2% to 4.53%) across different scanner field strengths, manufacturers, and models (Fig 3).

### Repeatability and reproducibility of human measurements

**Fig 4:**
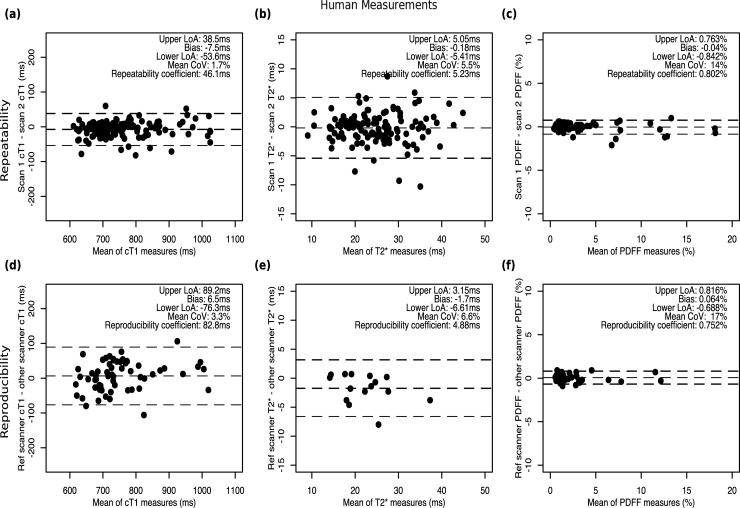
Repeatability and reproducibility of human multiparametric MRI measurements. Bland-Altman plots from human measurements across manufacturer and field strength for **(a)** cT1, **(b)** T2*, and **(c)** PDFF.

Standardized cT1 in participants demonstrated high repeatability (CoV 1.7%, bias -7.5 ms, 95% LoA of -53.6 to 38.5 ms) and reproducibility (CoV 3.3%, bias 6.5 ms, 95% LoA of -76.3 ms to 89.2 ms) across different scanner field strengths, manufacturers, and models. T2*-mappings showed good repeatability (CoV 5.5%, bias -0.18 ms, 95% LoA of -5.4 to 5.1 ms) and reproducibility (CoV 6.6%; bias -1.7ms; 95% LoA of -6.6ms to 3.2 ms). Similarly, PDFF measurements also showed good repeatability (CoV 14%, bias -0.04%, 95% LoA of -0.84 to 0.76%) and reproducibility (CoV 17%, bias 0.06%, 95% LoA of -0.69 to 0.82%) across different scanner field strengths, manufacturers, and models (Fig 4).

To interrogate the cT1 metric further, a random-effects model was generated to determine the variation that could be accounted for by each explanatory variable: scanner type (Avanto^fit^ 1.5T, Prisma 3T, Skyra 3T, Ingenia 1.5T, Ingenia 3T), scan repeat (SR1, SR2), analyst (AN1, AN2), and analysis repeat (AR1, AR2). Inspection of the model indicated that most of the variance in cT1 could be accounted for by study participants (ICC = 0.91), with minimal contribution from the other explanatory variables (scanner type = 0.04, scan repeat = 0.003, analyst = 0, analysis repeat = 0, residual = 0.05).

## Discussion

The primary goal of this study was to systematically test the repeatability and reproducibility of multiparametric-MRI derived measurements across scanner field strength, manufacturer and model in human participants and phantoms. We report the overall repeatability and reproducibility of standardised cT1, T2*, and PDFF measurements.

High repeatability and reproducibility was demonstrated in each metric tested. We report a 3.3% CoV in cT1 measurements across different manufacturer, field strength, and scanner model combinations on 61 participants who had mixed liver disease aetiology as well as those without any history of liver disease to represent the wide range of physiological values in the population. Interrogation of the cT1 metric indicated that most of the variance could be accounted for by study participants (ICC = 0.91), with minimal contributions from scanner type and scan repeat, further supporting the high reproducibility of this measurement.

In a recent study, Harrison and colleagues [23] reported repeatability of cT1, MR Elastography (MRE), and shear-wave ultrasonic elastography (LSM) to reveal CoVs of 3.1%, 11%, and 40% respectively. Similarly, Trout and colleagues [24] reported an average of 10.7% CoV in liver stiffness measurements across different manufacturer, field strength, and sequence combinations on 24 healthy adult volunteers with MRE [24]. However, it is not possible to compare the precision performance of these methods using CoV alone, as the underlying physiological properties and clinically-relevant dynamic range of the techniques are different, and in the Trout and colleagues study, subjects with known liver disease were not included.

Hines et al [25] reported that liver stiffness measurements from MRE varied by 8% between examinations in the same patient performed on the same day, and this increased to 12% when examined on different days separated by 2–4 weeks. In our study same scanner repeatability measurements were performed on the same day and reproducibility on different scanners were performed either on the same day or up to 1-week in between. It is possible that the short time period between serial examinations may have led to an underestimation of physiological variability and consequently a narrower cT1 range within subjects, and it is possible that this may increase with intermediate (e.g. 1 week) and longer (e.g. 6-months) time intervals. Future investigations could define within-subject variability in cT1 measurements to characterise longitudinal fluctuations in this metric.

In a recent study, Bane and colleagues [26] tested T1 repeatability and reproducibility in a T1 phantom across 10 MRI scanners. Using an optimized inversion recovery spin echo technique, they report a median repeatability CoV of 0.3%, and reproducibility CoV of 8.21% at 1.5T and 5.46% at 3T. One site in that study also ran a MOLLI experiment as in this study; the repeatability CoV was reported at 0.68% with a standard error of 4.64%.

PDFF has been recognised as the best current metric for a standardised MR-based biomarker of tissue fat concentration [27]. A meta-analysis of pooled data collected from 28 published studies demonstrated high precision of MR-PDFF across different field strengths, manufacturers and reconstruction methods, with repeatability and reproducibility coefficients of 2.99% and 4.12% respectively [28]. We report a repeatability coefficient of 0.8% and reproducibility coefficient of 0.75%, indicating excellent precision of this metric, in line with the literature.

Although we recruited subjects with liver diseases and BMI up to 39, subjects only had liver fat up to about 18% PDFF. There is a known contribution of liver fat to the T1 measurement [29] that is strongly dependent on the readout parameters. Good inter-scanner reproducibility was demonstrated in this population with these parameters with no trend of worse reproducibility with increasing fat fraction, but it is possible that still higher liver fats would show worse reproducibility. Acquisition of MOLLI data with a fat suppression technique is only available on one scanner platform; therefore, similar data could not be taken to measure reproducibility across platform. Other limitations in this study include biases from more 1.5T than 3T phantom and in-vivo reproducibility data, the choice of reference scanner, and limited Philips data. Finally, the MOLLI based technique [17] for T1 mapping used here only sampled 1 slice in each breath-hold. This is a limitation of the readout method, rather than of the technique.

Due to practical limitations, only a small number of participants were evaluated using the Philips scanners at 1.5T and 3T. Although a more balanced sample size per scanner would have been preferable, multiple phantom measurements performed across these scanners showed excellent reproducibility. The ability to standardise across different scanner field strength, manufacturers, and models, is important in the clinical trial setting where accurate and consistent evaluation of key outcomes across treatment interventions and patient groups can be aided by the ability to compare data gathered from multiple sites.

## Conclusions

Multiparametric MR-derived metrics, cT1, T2* and PDFF, have good repeatability and reproducibility that can quantify liver tissue characteristics independent of scanner manufacturer (Philips or Siemens) and field strength (1.5T or 3T). Multiparametric MRI is a non-invasive method that does not require additional hardware, and can be completed in less than 15-minutes, which will have important implications for routine monitoring and assessment of the liver in clinical practice. The ability to standardize metrics will be important in the clinical trial settings for evaluating treatment interventions.

## Supporting information

S1 File MRI scanning sequences. (DOCX)

S2 File T1-mapping Functions and precision. (DOCX)

## Abbreviations

CoV: Coefficient of Variation
cT1: corrected T1
ICC: Intraclass correlation coefficient
LoA: Limits of Agreement
MRI: Magnetic Resonance Imaging
PDFF: Proton Density Fat Fraction
