# Supplementary material for: Repeatability and reproducibility of multiparametric magnetic resonance imaging of the liver
Source: PLoS One. 2019 Apr 10;14(4):e0214921. doi: 10.1371/journal.pone.0214921 (PMC6457552; doi:10.1371/journal.pone.0214921)
Supplement: S1 File — (DOCX) [file pone.0214921.s001.docx]

## S1. File MRI scanning sequences

*Siemens 3T MRI Sequences*

Used on Siemens Skyra 3T (syngo MR E11A), and Siemens Prisma 3T (E11C) scanners:

1. *shMOLLI (Myomaps):* 192x144 acquisition matrix, segments=84, bandwidth=965 Hz/Pixel, Oversampling=0, Partial Fourier=6/8 (75%), Parallel imaging factor=2, 8mm slice thickness, flip angle=35°, FOV=440mm x 330mm, echo spacing (SSFP TR) =2.43ms, TE=1.05ms, initial TI=100ms, TI increment= 80ms.
2. *T2star and Dixon:* 128x104 acquisition matrix, 6mm slices thickness, flip angle=20°, FOV=400mmx325mm, TE = 1.23ms, echo spacing=1.23ms, number of echoes = 8, even echoes were used for fitting T2* and echoes 2, 3 & 4 were used for *Dixon* processing.

*Siemens 1.5T MRI Sequences*

Used on Siemens Avanto^fit^ 1.5T (syngo MR E11C) scanners:

1. *shMOLLI (Myomaps):* 192x144 acquisition matrix, segments=84, bandwidth=246 Hz/Pixel, Oversampling=0, Partial Fourier=6/8 (75%), Parallel imaging factor=2, 8mm slice thickness, flip angle=35°, FOV=440mm x 330mm, echo spacing (SSFP TR) =4.76ms, TE=1.93ms, initial TI=145ms, TI increment = 80ms.
2. *T2star and Dixon:* 128x104 acquisition matrix, 6mm slices thickness, flip angle=20°, FOV=400mmx325mm, TE = 2.38ms, echo spacing=2.38ms, number of echoes = 8, even echoes were used for fitting T2* and echoes 2, 3 & 4 were used for *Dixon* processing.

*Philips 3T MRI Sequences*

Used on Philips Ingenia 3T (5.3.0) scanners:

1. *MOLLI (CardicQuant):* 192x144 acquisition matrix, TFE factor=54, bandwidth=957.4 Hz/Pixel, Scan percentage=100%, Partial Fourier=6/8 (75%), Parallel imaging factor=2, 8mm slice thickness, flip angle=35°, FOV=440mm x 330mm, echo spacing (SSFP TR) =2.42ms, TE=1.20ms, initial TI=80ms, TI increment= 80ms.
2. *T2star and Dixon:* 128x108 acquisition matrix, 6mm slices thickness, flip angle=20°, FOV=400mmx337.5mm, TE = 1.19ms, echo spacing=1.19ms, number of echoes = 8, even echoes were used for fitting T2* and echoes 2, 3 & 4 were used for *Dixon* processing.

*Philips 1.5T MRI Sequences*

Used on Philips Ingenia 1.5T (5.3.0) scanners:

1. *MOLLI (CardicQuant):* 192x144 acquisition matrix, TFE factor=54, bandwidth=311.5 Hz/Pixel, Scan percentage=100%, Partial Fourier=6/8 (75%), Parallel imaging factor=2, 8mm slice thickness, flip angle=35°, FOV=440mm x 330mm, echo spacing (SSFP TR)=4.76ms, TE=1.93ms, initial TI=140ms, TI increment= 80ms.
2. *T2star and Dixon:* 128x104 acquisition matrix, 6mm slices thickness, flip angle=20°, FOV=400mmx337.5mm, TE = 2.37ms, echo spacing=2.37ms, number of echoes = 8, even echoes were used for fitting T2* and echoes 2, 3 & 4 were used for *Dixon* processing.
