# Supplementary material for: Repeatability and reproducibility of multiparametric magnetic resonance imaging of the liver
Source: PLoS One. 2019 Apr 10;14(4):e0214921. doi: 10.1371/journal.pone.0214921 (PMC6457552; doi:10.1371/journal.pone.0214921)
Supplement: S2 File — (DOCX) [file pone.0214921.s002.docx]

## *S2. File T1-mapping Functions and precision*

T1-mapping functions were derived from independent phantom measurements and have the form:

$$T_{1}=m.T_{1 Philips}+c$$

1. *Mapping Philips Ingenia 3T to Siemens Prisma 3T
   m = 0.989, c = -4.83 ms*
2. *Mapping Philips Ingenia 1.5T to Siemens Avanto 1.5T
   m = 0.924, c = 23.1 ms*

*T1 precision*

In vivo precision of native T1 had a repeatability of [CoV 1.7%, bias -6.8ms, 95% LoA of -46.6 to 33ms] and reproducibility of [CoV 5.7%, bias -25ms, 95% LoA of -136.0 to 86.6ms]. Using the inter-scanner model mapping gave a reproducibility of [CoV 4.9%, bias -14ms, 95% LoA of -116.0 to 88.2ms]. As liver T1 differs between 1.5T and 3T, only within-field strength comparisons were made for reproducibility. Inter-scanner mappings are not applicable to repeatability. These results for T1 and inter-scanner mapped T1 are similar to the precision of the inter-field strength corrected cT1.
